# A novel closed chest drainage device

**DOI:** 10.3389/fmed.2025.1675836

**Published:** 2025-10-17

**Authors:** Shaoqing Huang, Xu Song, Qiang Shi, Jie Li

**Affiliations:** Department of Thoracic Surgery, Ningbo No. 2 Hospital, Ningbo, China

**Keywords:** lung surgery, negative pressure drainage device, novel closed chest drainage device, digital drainage device, traditional closed chest drainage systems

## Abstract

**Purpose:**

Negative pressure closed thoracic drainage improves drainage efficiency, accelerates the removal of fluid and air, and facilitates lung re-expansion. Conventional wall negative pressure systems restrict patient mobility, while digital drainage devices are associated with high costs that hinder widespread clinical adoption. Hence, there is an urgent need for a cost-effective, practical, and portable negative pressure drainage system.

**Methods:**

Based on the principles outlined in the Chinese national patent (ZL 202122505281.4), we innovatively integrated a micro-pump and power system into a compact, portable negative pressure generator, which was then connected to the pressure-regulating chamber of a conventional three-chamber closed drainage bottle. This integration resulted in a novel portable closed thoracic drainage device with active negative pressure control. The device underwent *in vitro* testing followed by preliminary proof-of-concept evaluation.

**Results:**

The test results indicate that the novel closed thoracic drainage device can achieve a maximum negative pressure of approximately 20 cm H₂O and a maximum airflow rate of 15 L/min. The novel device was initially used in three patients.

**Conclusion:**

A novel type of negative pressure closed thoracic drainage device has been successfully developed and a preliminary concept verification has been carried out. This device offers the advantages of cost-effectiveness and portability, demonstrating potential for wide application in postoperative thoracic drainage following lung surgery.

## Introduction

**Figure 1:**
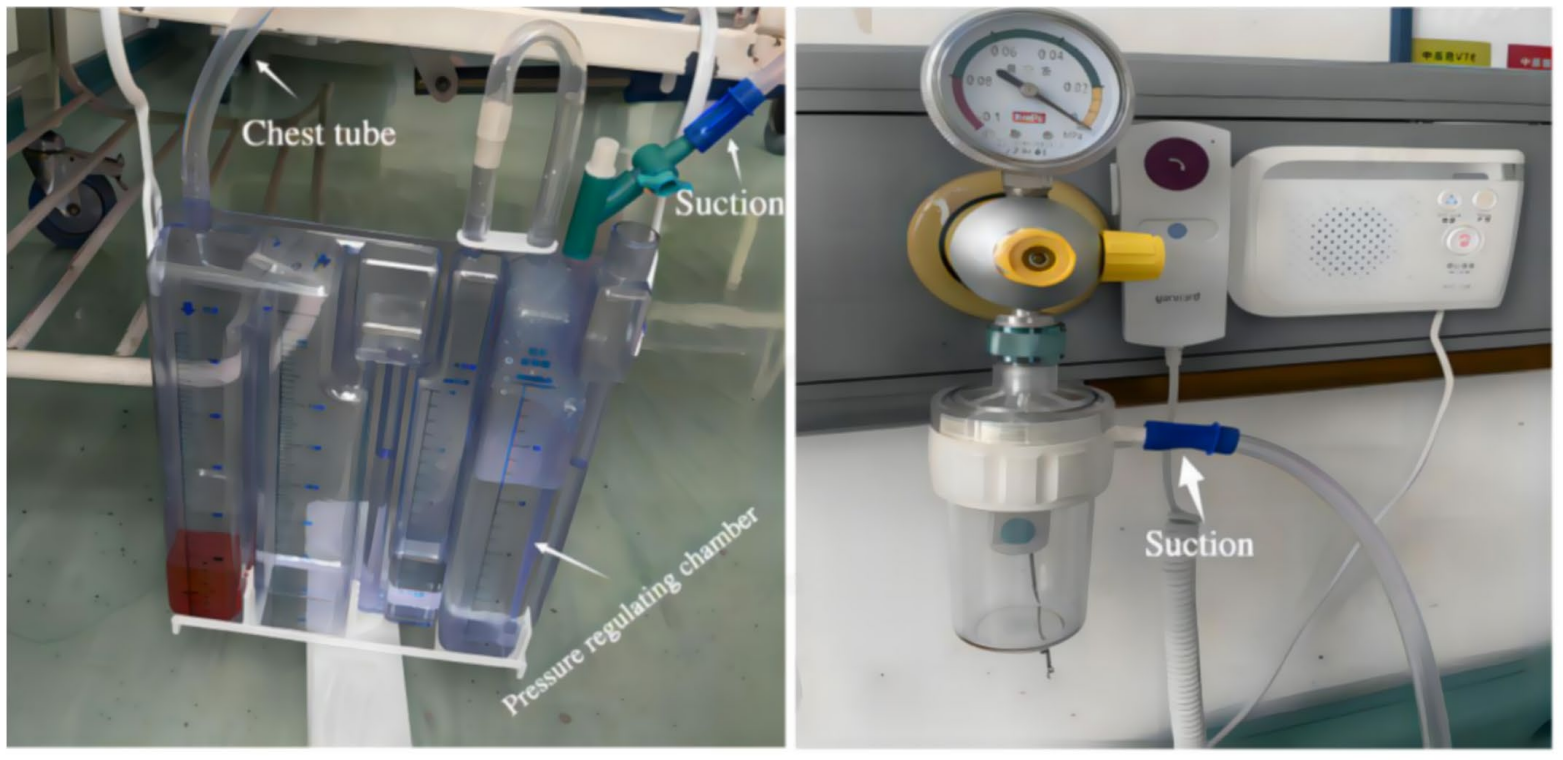
Wall suction drainage system.

The primary function of closed thoracic drainage is to effectively remove air and fluid from the pleural cavity, thereby facilitating lung re-expansion. As a result, it has become an essential component of perioperative management in thoracic surgery (1). Following lung surgery, closed thoracic drainage is generally categorized into two types: simple water-seal drainage and negative pressure drainage (2). Although clinical practice regarding postoperative thoracic drainage remains controversial, accumulating evidence supports the advantages of negative pressure drainage, including earlier chest tube removal and shorter postoperative hospital stays (3–5). In 2017, postoperative negative pressure drainage following lung surgery was incorporated into translational medicine clinical guidelines. However, conventional three-chamber closed thoracic drainage systems connected to wall suction units restrict patient mobility (Figure 1). Digital drainage systems, although advanced, are costly and require changes to established surgeon practices, limiting their widespread clinical adoption (6, 7). Inspired by a breast pump used at home, we previously proposed a novel portable negative pressure closed thoracic drainage device to overcome the limitations of traditional wall-mounted systems and explore its potential benefits (6). In this study, we integrated a micro-pump and power supply system into a modified closed thoracic drainage setup by connecting it to the pressure-regulating chamber of a standard three-chamber drainage bottle, thereby developing a novel type of split-design negative pressure thoracic drainage device. This device is cost-effective, practical, and portable. Its initial clinical application has been undertaken in a preliminary setting.

## Methods

Device description and preliminary use cases.

### Device description

**Figure 2:**
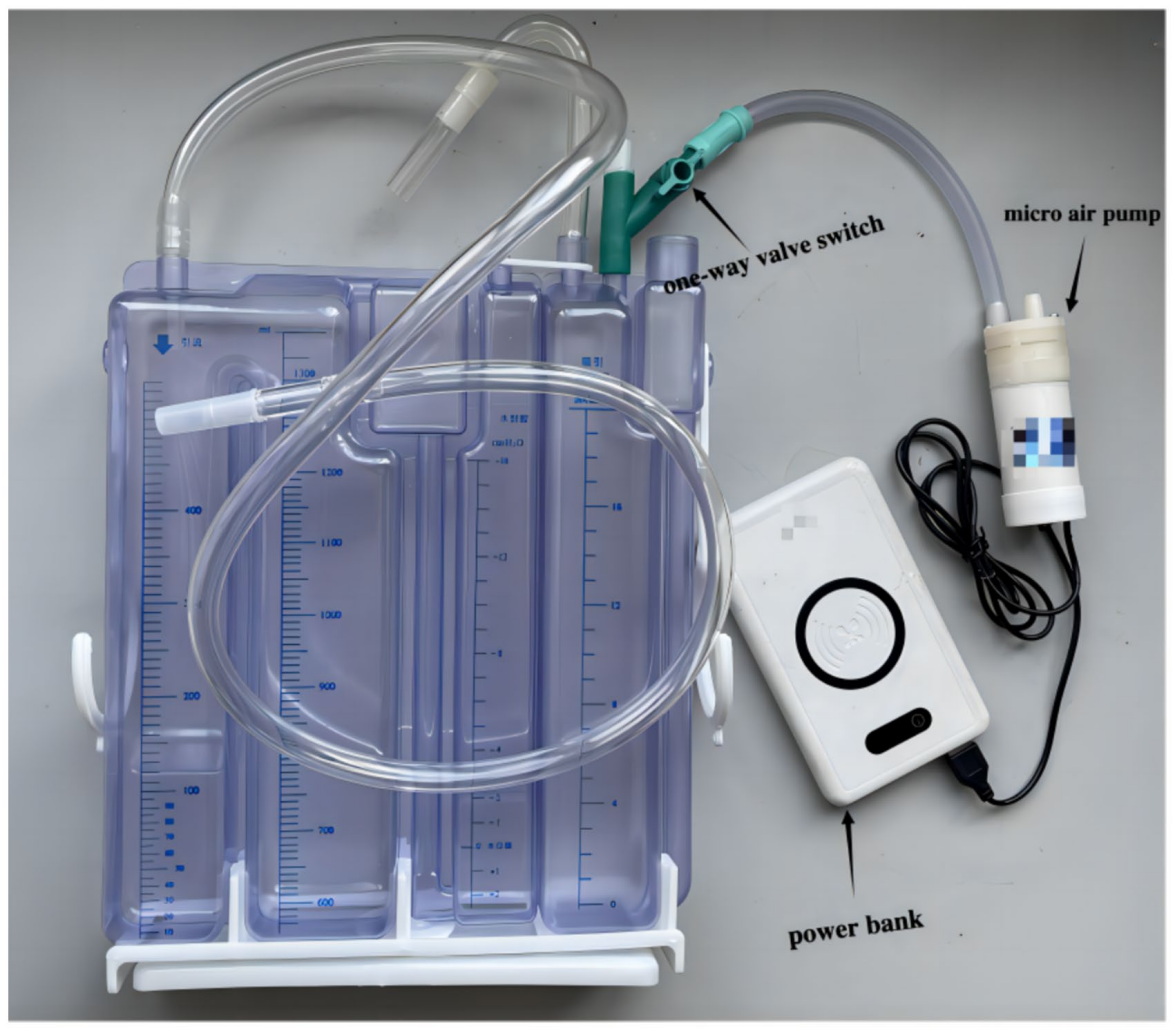
The novel closed chest drainage device.

The novel closed chest drainage device consists of a three-chamber closed drainage bottle, a micro air pump and a power bank (Figure 2). Comparison of different negative pressure drainage systems is presented in Table 1.

**Table 1:** Comparison of negative pressure systems.

Device type	Cost	Portability	Leak monitoring	Data logging	Noise	Clinical use
Wall suction	Low	No	No	No	Yes	Quiet widely used
Digital system	High ($2000–$3,000)	Yes	Yes	Yes	No	Quiet limited by cost
Novel device	Very low ($20)	Yes	No	No	Yes	Prototype stage

#### Three-chamber closed drainage bottle

Made in China; About 6 dollars; The three-chamber closed drainage bottle has a capacity cavity, a water sealing cavity, and a pressure regulating cavity. The maximum height of the water column in the pressure regulating chamber is 20 cm; The biggest chest fluid storage capacity is more than 1,300 mL. The pressure regulating chamber has a one-way valve, which can be used to adjust the flow rate of gas by turning the valve switch.

#### Micro air pump

Made in China; About 6 dollars; Micro air pump has an air inlet and outlet, The inner diameter of the pipe to 8 mm; Flow 15 L/ min; USB input type. There is a start-stop switch at the top.

#### Power bank

Made in China; about 8 dollars; capacity 20,000 ma; USB output type.

#### Preliminary use cases

The novel device has undergone proof-of-concept testing; however, the findings are limited by the small sample size due to the limited number of clinical cases. Three patients who underwent lobectomy, segmentectomy, and wedge resection, respectively, were enrolled in the study. Baseline clinical information was collected preoperatively (Table 2). The novel drainage device was implemented immediately after surgery, with negative pressure adjusted to −20 cm H₂O to maintain 3–5 bubbles per second in the pressure-regulating chamber via the single-valve switch. Negative pressure suction was maintained for 24 to 48 h or until no bubbles appeared in the water-seal chamber during coughing. Power supply was ensured by replacing the portable power bank during use; wall suction was used when patients remained in bed. Continuous drainage was maintained if air bubbles were observed in the water-seal chamber upon coughing. Postoperatively, all patients received routine intravenous infusion of non-steroidal analgesics twice daily. Additional analgesic injections were administered as needed for breakthrough pain. Chest tube removal was performed after 24 h of bubble-free drainage during coughing and when pleural fluid became lightly serosanguineous. A routine chest X-ray was obtained 24 h after tube removal. One week after discharge, patients returned for follow-up chest X-ray to assess for residual pneumothorax or pleural effusion, with symptomatic management provided if necessary. Daily and total pleural fluid volumes were recorded, along with the timing of chest tube removal and hospital discharge.

**Table 2:** Basic information of the three patients.

Gender	Age(y)	Smoking	FEV1(%)	Diameter of tumor(cm)	Type of Surgery	pathology
Female	56	No	86	0.7	Uniportal VATS wedge resection of the right upper lobe + lymph node sampling	Benign
Male	65	No	96.7	0.8	Uniportal VATS lingual resection of the left upper lobe + pleural adhesion release + lymph node dissection	Adenocarcinoma
Female	74	No	72.5	1.0	Uniportal VATS wedge resection of right upper lobe + right middle lobe resection + lymph node dissection + pleura release	Adenocarcinoma

## Results

**Figure 3:**
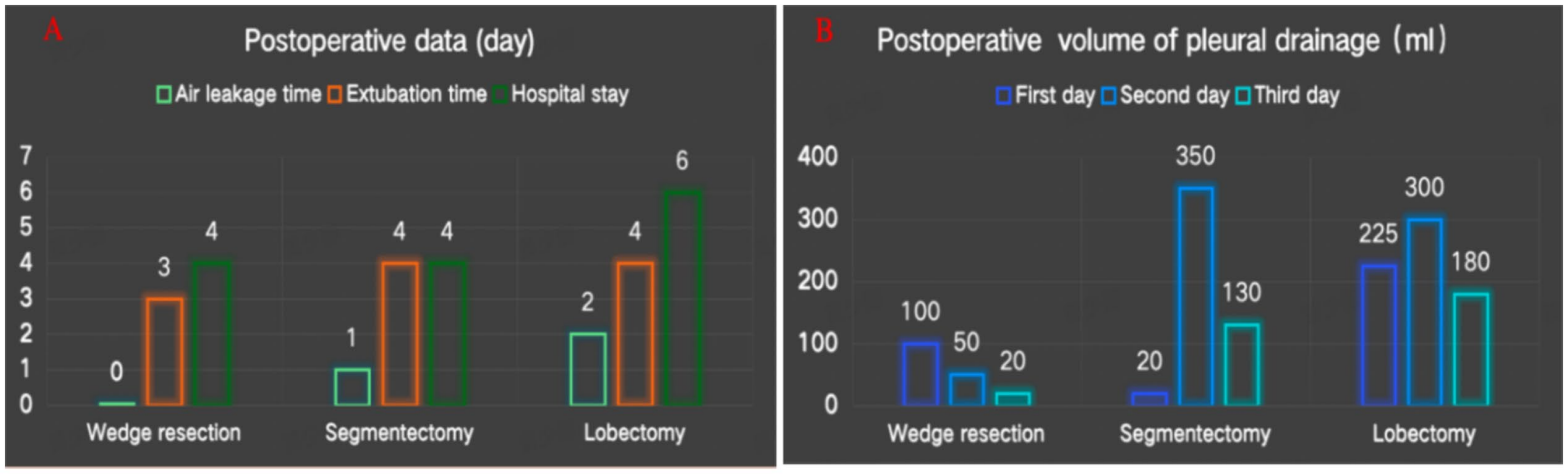
**(A)** Postoperative data. **(B)** Postoperative volume of pleural drainage.

The test results indicate that the novel closed thoracic drainage device can achieve a maximum negative pressure of approximately 20 cm H₂O and a maximum airflow rate of 15 L/min. The average duration of postoperative air leakage was 1 day, the mean time to chest tube removal was 3.7 days, and the average postoperative hospital stay was 4.7 days (Figure 3A). The mean daily postoperative chest drainage volume was 124 mL (Figure 3B). The device was successfully applied in all three patients, who reported no discomfort, recovered well, and were discharged uneventfully. One week after discharge, follow-up chest X-rays showed no evidence of pleural effusion or pneumothorax in any of the patients.

## Discussion

**Figure 4:**
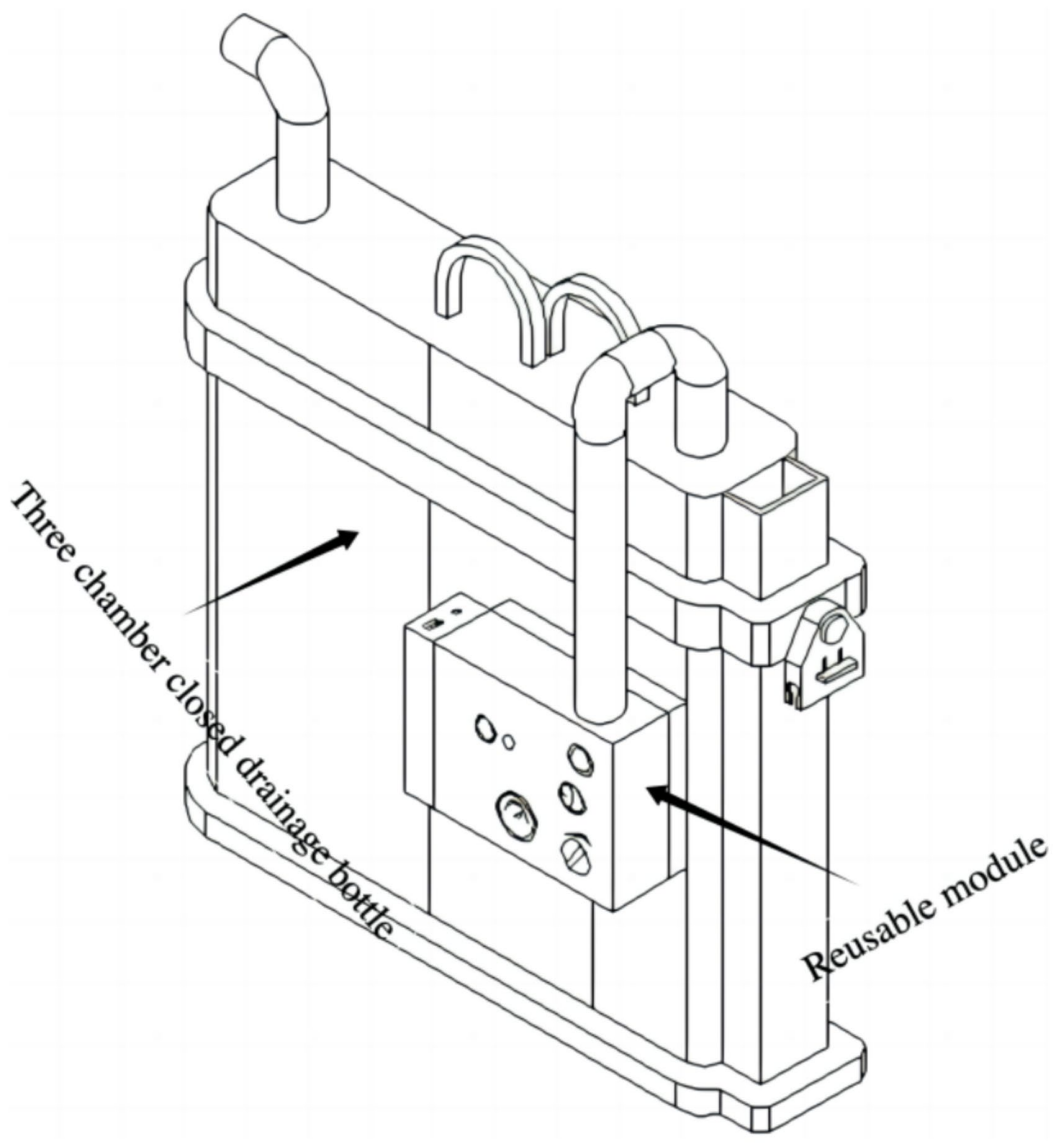
The novel closed chest drainage device.

In the perioperative management of chest tubes during pulmonary surgery, closed thoracic drainage devices play a crucial role, and negative pressure drainage has been shown to facilitate postoperative recovery (3–5). Traditional closed thoracic drainage systems include single-chamber, double-chamber, and triple-chamber drainage bottles. Based on their operating principles, these devices can be classified into wet and dry closed thoracic drainage systems (8). Conventionally, standard triple-chamber closed drainage bottles require connection to wall suction to generate negative pressure, which often restricts patient mobility in the postoperative period. Although dry digital drainage systems overcome this limitation, they are costly—typically priced between $2,000 and $3,000—and may disrupt established surgeon workflows. Moreover, despite their ability to monitor air leaks, digital systems do not consistently lead to earlier chest tube removal, suggesting limited surgeon confidence in their reliability for air leak assessment (9). Recently, Le et al. introduced a hybrid closed thoracic drainage device that combines a simple water seal with a digital system, indicating its potential for managing complex persistent pneumothorax; however, cost information was not reported (10). We initially proposed the concept of a closed thoracic drainage bottle integrated with a miniature air pump for negative pressure drainage, as described in a Chinese national patent (ZL 202122505281.4). Subsequently, in our prior study, we refined this concept into an integrated portable negative pressure drainage device and discussed its advantages and limitations (6, 7). In the present study, we innovatively assembled a miniature air pump and power system and connected them to the pressure-regulating chamber of a conventional triple-chamber closed drainage bottle, thereby developing a novel split-type negative pressure triple-chamber drainage device (Figure 4). A preliminary proof-of-concept evaluation was also conducted.

The design offers several key advantages. First, it builds upon the clinically validated triple-chamber drainage bottle; thus, if the active negative pressure system fails, the device reverts to standard passive drainage function, enhancing both safety and clinical feasibility. Second, the device preserves established clinical practices—for example, surgeons continue to assess air leaks by observing bubble formation in the water-seal chamber—minimizing the need for behavioral adaptation and facilitating clinical adoption. Third, the modular (split) design allows for future cost reduction: the triple-chamber bottle can be disposable, while the negative pressure generation module (pump and power supply) can be reused. This low-cost, reusable model significantly enhances the potential for large-scale clinical implementation.

However, this study has limitations. The device cannot objectively quantify air leak parameters, lacks data traceability, has a limited maximum negative pressure capacity, and generates operational noise. Additionally, the current findings are based on a small sample size, limiting generalizability.

Despite these drawbacks, clinical experience suggests that reliable negative pressure support—rather than digital monitoring—is the primary need for clinicians and patients, particularly given that clinical decisions are typically made during routine morning rounds.

Looking ahead, we plan to conduct larger-scale clinical trials to further evaluate the device’s performance and develop a remote monitoring system incorporating a sensor module capable of collecting and transmitting clinical data in real time, enabling continuous assessment of patient recovery and supporting personalized care planning.

In conclusion, we have developed a novel portable negative pressure thoracic drainage device that preserves existing surgeon workflows and offers significant cost advantages, demonstrating strong potential for widespread clinical application.

## Data Availability

The original contributions presented in the study are included in the article/supplementary material, further inquiries can be directed to the corresponding authors.
